# The Association of Ageism, COPD-related Stigma, and Psychosocial Health in Chronic Obstructive Pulmonary Disease

**DOI:** 10.1093/geroni/igaf122.2212

**Published:** 2025-12-31

**Authors:** Seoyoon Woo, Jeonghwa Lee, Susan Glose, Yeoun Soo Kim-Godwin

**Affiliations:** University of North Carolina Wilmington, Wilmington, North Carolina, United States; The University of North Carolina at Wilmington, Wilmington, North Carolina, United States; The University of North Carolina at Wilmington, Wilmington, North Carolina, United States; The University of North Carolina at Wilmington, Wilmington, North Carolina, United States

## Abstract

**Rationale/Objectives:**

People with chronic obstructive pulmonary disease (COPD) may experience an accelerated aging process that affects both their health and social roles. This study aimed to examine the relationship between stigma related to aging and that associated with COPD, and the psychosocial health of individuals living with COPD.

**Methods:**

A cross-sectional study using an online survey method was conducted. Participants who self-reported having COPD (N = 101; mean=61.4 years) completed a series of questionnaires, including the Everyday Ageism Scale, the COPD-related Stigma Scale, the Attitudes to Aging Scale (AAQ), the PROMIS Anxiety (SF-8a), the Social Isolation Scale, and questions on unplanned doctor visits due to acute exacerbations, and age. ANOVA and multivariate regression models were used to analyze the data.

**Results:**

There were no significant differences in ageism (mean=14.65±5.13) and COPD-related stigma (mean=2.37±0.57) among different age groups. Using Wilks’ criterion, the combined ageism and COPD-related stigma were significantly associated with the following factors: the AAQ-psychosocial loss scale (F(2, 94)=9.75, p < 0.001; partial η^2=0.17), anxiety (F(2, 94)=4.26, p = 0.017; partial η^2=0.08), social isolation (F(2, 94)=4.21, p = 0.018; partial η^2=0.08), and acute exacerbation episodes (F(2, 94)=4.89, p = 0.010; partial η^2=0.09). However, there was no significant relationship with age (F(2, 94)=2.42, p = 0.094; partial η^2=0.05). Univariate analyses indicated that anxiety and age were significantly associated with ageism, but not with COPD-related stigma. Conversely, social isolation was significantly associated with COPD-related stigma, but not with ageism.

**Conclusion:**

Findings enhance the understanding of the relationships between aging-related and COPD-related stigma experiences and psychosocial factors in individuals with COPD.

